# Host Genetic Constraints on the Horizontal Transmission of *Daphnia*-associated Microbiota

**DOI:** 10.1264/jsme2.ME26003

**Published:** 2026-05-27

**Authors:** Ryotaro Ichige, Jotaro Urabe

**Affiliations:** 1 Graduate School of Life Sciences, Tohoku University, 6–3 Aoba, Aramaki, Aoba-ku, Sendai, 980–8578, Japan

**Keywords:** *Daphnia* cf. *pulex*, host genetic effects, gut and epidermal bacteria, horizontal transfer, symbiotic microbiota

## Abstract

The taxonomic composition of *Daphnia* microbiota is affected not only by external environmental conditions, but also by the host’s internal physiological state, which is partly governed by genetic factors. However, the extent to which host genetics constrain the composition of associated bacterial communities remains unclear. In the present study, we conducted mixed-culture experiments using obligately parthenogenetic *Daphnia* cf. *pulex* individuals from genetically distinct lineages. The results obtained showed that the taxonomic composition of host-associated microbiota significantly differed between genotypes, both within and across lineages, with certain bacterial taxa being exclusive to specific genotypes. When genetically distinct hosts were co-cultured, some bacterial taxa initially exclusive to one genotype appeared in the microbiota of another, indicating the horizontal transmission of microbiota between hosts. Nevertheless, the overall taxonomic composition of microbiota was largely unaffected by the presence of genetically different hosts. These results suggest that although the horizontal transfer of microbiota occurs between different *Daphnia* genotypes, it is not extensive enough to override genotype-specific microbiota compositions. Therefore, in *D.* cf. *pulex*, host genetics play a major role in shaping the composition of the associated microbiota.

Animals host diverse microbial communities on their epidermis and within their gastrointestinal tracts, collectively termed the host-associated microbiota. These communities play pivotal roles in host nutrition (Flint *et al.*, 2012), metabolism (Koh and Bäckhed, 2020), immunity (Ahern and Maloy, 2020), and fitness-related traits, such as growth and reproduction (Peerakietkhajorn *et al.*, 2015). Recent studies across various taxa—including primates (Flynn *et al.*, 2022), fish (Sipkema *et al.*, 2015; Sylvain and Derome, 2017), birds (Davidson *et al.*, 2021), and insects (Juma *et al.*, 2021)—demonstrated that the compositions of host-associated bacterial communities are affected not only by external environmental conditions, but also by the host’s internal physiological state, which is at least partially governed by genetic factors. Furthermore, evidence indicates that even closely related species harbor distinct microbiota, underscoring the species-specific genetic regulation of microbial assemblages (Houwenhuyse *et al.*, 2024). However, the extent to which intraspecific variations in microbiota compositions is governed by host genetics remains unclear.

In aquatic ecosystems, *Daphnia* species serve as critical intermediaries, channeling energy from primary producers to higher trophic levels (Lampert and Sommer, 2007). Under favorable environmental conditions, they reproduce asexually, and this parthenogenetic reproduction ensures genetic consistency across generations, making them ideal models for investigating genetic constraints on microbiota compositions (Peerakietkhajorn *et al.*, 2015; Zhang *et al.*, 2021; Gurung *et al.*, 2024). Due to both their ecological importance and biological relevance, microbiota associated with *Daphnia* have been extensively exami­ned. Notably, certain bacterial taxa have been shown to modulate *Daphnia* growth and reproduction (Peerakietkhajorn *et al.*, 2015; Zhang *et al.*, 2021). Moreover, the taxonomic compositions of *Daphnia*-associated microbiota vary depending on the host’s physiological condition and genotype (Ichige and Urabe, 2023). Recent findings suggest that priority effects, in which early colonizing microbes play a role in the establishment of subsequent microbial taxa (Fukami, 2015; De Meester *et al.*, 2016), also contribute to shaping these communities, potentially under the regulation of the host genotype (Gurung *et al.*, 2024). If the compositions of host-associated microbiota are governed by host genetic traits, the horizontal transfer of these microbes between genetically distant individuals—even within the same species—may be limited. Nevertheless, few studies have explicitly exami­ned horizontal transfer among *Daphnia* individuals.

Several phylogenetically distinct lineages of *Daphnia* cf. *pulex*, introduced from North America, have been identified in Japan (So *et al.*, 2015). Among these, the JPN1 and JPN2 lineages include multiple genotypes that exhibit genetic divergence within each lineage (Ohtsuki *et al.*, 2022). These genotypes reproduce obligately via parthenogenesis, precluding sexual recombination even when they occur in sympatry (So *et al.*, 2015; Ohtsuki *et al.*, 2022). Additionally, ecological traits differ to some extent between the genotypes of the JPN1 and JPN2 lineages (Tian *et al.*, 2024). If microbiota compositions consistently differ among genotypes from different lineages, even when co-cultured under identical environmental conditions, these differences may reflect host genetic constraints. This suggests a limited likelihood of horizontal microbiota transfer between genetically distinct hosts, even within the same species.

In the present study, we investigated the extent of horizontal transfer and the genetic constraints on host-associated microbiota in *Daphnia* cf. *pulex* using genetically distinct genotypes from the JPN1 and JPN2 lineages. Specifically, we conducted mixed-culture experiments in which genotypes from different lineages were co-cultured in a shared environment. We then performed metagenomic anal­yses to compare the microbiota of host individuals reared in isolation with those reared alongside genetically different genotypes. Using this approach, we evaluated both the degree of horizontal microbiota transfer and the effects of host genetic backgrounds on the structure of associated microbial communities.

## Materials and Methods

### Experimental organisms

The genotypes used in this study included A1 and A5 from the‍ ‍*Daphnia* cf. *pulex* JPN1 lineage, and C and G from the JPN2 lineage, all originally collected in Japan. Genotypes A1 and G were collected from Lake Hataya Ohnuma in Yamagata Prefecture, genotype A5 was collected from Furuichi Oike Pond in Tottori Prefecture, and genotype C was collected from Fukami Ike Pond in Nagano Prefecture (So *et al.*, 2015; Tian *et al.*, 2024). These genotypes have been maintained under standardized laboratory conditions for more than five years at 20°C in COMBO medium (Kilham *et al.*, 1998), with the green alga *Scenedesmus* sp. being provided as a food source (Tian *et al.*, 2024). These algae were cultured in an axenic flow-through system with a daily dilution rate of 0.5 L using COMBO medium. Cells were harvested and counted under an optical microscope (Olympus). Based on the previously measured cell-specific carbon content of this alga (2.09×10^–8^ mg C cell^–1^ Tian *et al.*, 2019), we calculated the amount of algae required to achieve the target carbon concentration and used this to feed experimental animals. Five to seven juvenile females of each genotype were reared together in 500 mL of COMBO medium supplemented with green algae at a concentration of 1 mg CL^–1^. The medium was refreshed every other day, and animals were monitored daily for the production of neonates. After a minimum of 7 days, neonates born within a 24-h window were collected and used in experiments.

### Experiment design

Four neonates from each of the four genotypes were placed into each of two polycarbonate bottles containing 250 mL of COMBO medium supplemented with 1 mg CL^–1^ of green algae cultured under sterile conditions. These served as the control treatments. In the mixed treatments, two neonates from a JPN1 genotype (A1 or A5) and two from a JPN2 genotype (C or G) were co-cultured in each of three polycarbonate bottles filled with 250 mL of the same algal food and medium (Table 1). Four inter-lineage genotype pairs were established: JPN1-A1 with JPN2-C, JPN1-A1 with JPN2-G, JPN1-A5 with JPN2-C, and JPN1-A5 with JPN2-G. Each pair was made in three replicate bottles. All control (*n*=2 per genotype) and mixed-treatment (*n*=3 per genotype pair) bottles were maintained in an incubator at 20°C under dim lighting. Animals were transferred to fresh COMBO medium with 1 mg CL^–1^ of algal food every 2 days using sterilized pipettes specific to each bottle to avoid cross-contamination. On day 8, all individuals were collected, placed into sterilized Eppendorf tubes, and stored at –24°C for further metagenomic anal­yses.

### DNA extraction from *Daphnia*

DNA extraction was performed following the protocol described by Ichige and Urabe (2021), with slight modifications. Briefly, an enzymatic lysis buffer was prepared by combining 3.6 mg lysozyme powder, 3.6 μL of 1 M Tris-HCl (pH 8.0), 0.72 μL of 0.5 M EDTA (pH 8.0), 21.6 μL of 10% polyoxyethylene (10) octylphenyl ether, and 154.08 μL of sterile distilled water. This buffer was added to Eppendorf tubes containing *Daphnia* individuals stored at –24°C. Specimens were homogenized using sterile pestles and incubated at 850 rpm for 1 h in a block bath shaker (MyBL-100CS, AS ONE) at 37°C, following the method of Sullam *et al.* (2018). DNA was then extracted using the DNeasy Blood and Tissue Kit (QIAGEN), yielding 200 μL of DNA extract per sample. Extracted DNA was used to genotype *Daphnia* and was also subjected to a metagenomic anal­ysis of host-associated microbiota.

### Genotyping of *Daphnia*

To identify the genotypes of *Daphnia* individuals in the mixed treatments, we employed a PCR-RFLP method. A 0.5-μL aliquot of extracted DNA was used to amplify a partial sequence of the mitochondrial NADH dehydrogenase subunit 5 (ND5) gene via PCR, following the protocol of So *et al.* (2015). The resulting amplicons were digested using two restriction enzymes, *Hpy*188III and *Mnl*I (New England BioLabs). Each 10-μL digestion reaction contained 5 μL of the PCR product, 2.9 μL distilled water, 1.0 μL Cresol Red, 1.0 μL 10× CutSmart Buffer, and 0.05 μL each of *Hpy*188III and *Mnl*I. The reaction mixture was incubated at 37°C for 2 h, followed by heat inactivation at 65°C for 20 min. We then applied the PCR-RFLP Reaction product to a 2% agarose gel (Agarose S; Nippon Gene) electrophoresed at 100 V for 30 min. After electrophoresis, the gel was stained with GelRed^TM^ (Biotium). The digested products were visualized under UV light, and genotype identification was performed based on characteristic fragment patterns: genotypes A1 and A5 (JPN1 lineage) yielded three fragments of 29, 147, and 630 bp, while genotypes C and G (JPN2 lineage) yielded three fragments of 29, 200, and 577 bp.

### Metagenomic anal­ysis of host-associated microbiota

The metagenomic sequencing of bacterial communities was performed simultaneously for all *Daphnia* individuals collected from both the control and mixed treatments. Whole-body DNA was extracted from each individual; therefore, the resulting metagenomic data included microbiota associated with both the gut and epidermis. In the control treatments, one or two individuals were selected from each of the two replicate bottles for each genotype. To balance the number of individuals exami­ned in the control treatments, four individuals per genotype were analyzed in each mixed treatment composed of JPN1-A1 and JPN2-C, and JPN1-A1 and JPN2-G. To select these individuals, we randomly selected one individual per genotype from each of the three replicate bottles. One additional individual per genotype was randomly selected from the remaining individuals across all three replicate bottles. In the mixed treatment composed of JPN1-A5 and JPN2-G, four individuals of JPN2-G were selected following the same procedure; however, for JPN1-A5, only three individuals (one from each bottle) were available and used (Table 2). In the mixed treatment composed of JPN1-A5 and JPN2-C, only one individual per genotype was available due to an accident; consequently, only one individual per genotype was analyzed for this treatment. Therefore, forty *Daphnia* individuals were processed for the metagenomic anal­ysis (Table 2).

In the first PCR, the V3–V4 region of the 16S rRNA gene was amplified using the primers 1^st^_341f_MIX (5′-*ACACTCTTTCCCTACACGACGCTCTTCCGATCT*-NNNNN-**CCTACGGGNGGCWGCAG**-3′) and 2^nd^_805r_MIX (5′-*GTGACTGGAGTTCAGACGTGTGCTCTTCCGATCT*-NNNNN-**GACTACHVGGGTATCTAATCC**-3′), where italicized sequences represent Illumina adapters, bold indicates 16S rRNA gene-specific regions, and ‘N’ denotes random nucleotides to increase sequence diversity. PCR reactions (total volume of 40 μL) consisted of 10 μL template DNA, 4 μL 10× Ex Taq^®^ buffer (TAKARA Bio), 3.2 μL dNTP mixture (2.5 mM each), 2 μL of each primer (10 μM), 0.4 μL TaKaRa Ex Taq^®^ Hot Start Version (TAKARA Bio), and 18.4 μL sterile distilled water. Thermal cycling conditions were as follows: initial denaturation at 94°C for 2 min; 35 cycles of 94°C for 30 s, 55°C for 30 s, and 72°C for 30 s; and a final extension at 72°C for 5 min. PCR products were purified using AMPure XP beads (Beckman Coulter) and eluted in 40 μL of 10 mM Tris-HCl (pH 8.0). Second-round PCR and sequencing were conducted by Bioengineering Lab. following the procedures outlined in Ichige and Urabe (2023).

### Data processing and statistical anal­yses

Bacterial 16S rRNA V3–V4 region sequence data were processed using QIIME 2 version 2022.8 (Bolyen *et al.*, 2019). Quality filtering, denoising, and the generation of amplicon sequence variants (ASVs) were performed using the DADA2 plugin (Callahan *et al.*, 2016). We obtained 1,222 ASVs with a minimum of 26,049 reads, a maximum of 40,740 reads, and an average of 31,450 reads. Sequences were aligned with MAFFT (Katoh *et al.*, 2019), and phylogenetic trees were constructed using FastTree2 (Price *et al.*, 2010). The taxonomic classification of ASVs was conducted using a naïve Bayesian classifier implemented in *scikit-learn* (Pedregosa *et al.*, 2011), trained on the SILVA 138 reference database (Quast *et al.*, 2013; Yilmaz *et al.*, 2014).

ASVs were primarily classified at the genus level; if genus-level resolution was not possible, classification was assigned at the family or order level. In downstream anal­yses, sequencing depth was normalized by rarefying all samples to 8,000 reads. Data integration and handling were performed in R version 4.3.2 (R Core Team, 2023). ASV, phylogenetic, and taxonomic information were imported into R as a phyloseq object using the qiime2R package (Bisanz, 2018), and metadata were organized using the phyloseq package (McMurdie and Holmes, 2013). To compare within-host ASV diversity among *Daphnia* genotypes, α-diversity was quantified for each individual using the Shannon index (Shannon, 1948), calculated from ASV read counts. Differences in the relative abundance of dominant bacterial taxa between the control and mixed treatments were assessed for each *Daphnia* lineage using the Wilcoxon rank-sum test.

To assess β-diversity, we calculated weighted UniFrac distances (WUD) (Lozupone *et al.*, 2011), which incorporate phylogenetic relationships among ASVs, and the Horn index (Horn, 1966), which considers quantitative differences in the composition, but not phylogeny of ASVs. These distance matrices were visualized via a principal coordinate anal­ysis (PCoA). The effects of the host lineage, genotype (nested within lineage), and treatment on β-diversity were tested using a three-way nested PERMANOVA with 9,999 permutations, implemented in the vegan package (Dixon, 2003).

We also performed variation partitioning to evaluate the relative contributions of the genotype and treatment to the observed microbiota composition using the *varpart* function in “vegan”, based on‍ ‍both the WUD and Horn indices. All data visualization and additional manipulations were performed using the R packages “phyloseq”, “tidyverse” (Wickham *et al.*, 2019), “sjPlot” (Lüdecke *et al.*, 2023), “cowplot” (Wilke, 2020), “ggpubr2” (Kassambara, 2023), “ggtree” (Yu *et al.*, 2017), “RColorBrewer” (Neuwirth, 2022), “speedyseq” (McLaren, 2023), and “microbiome” (Lahti and Shetty, 2023).

## Results

### α-Diversity

**Fig. 1.**
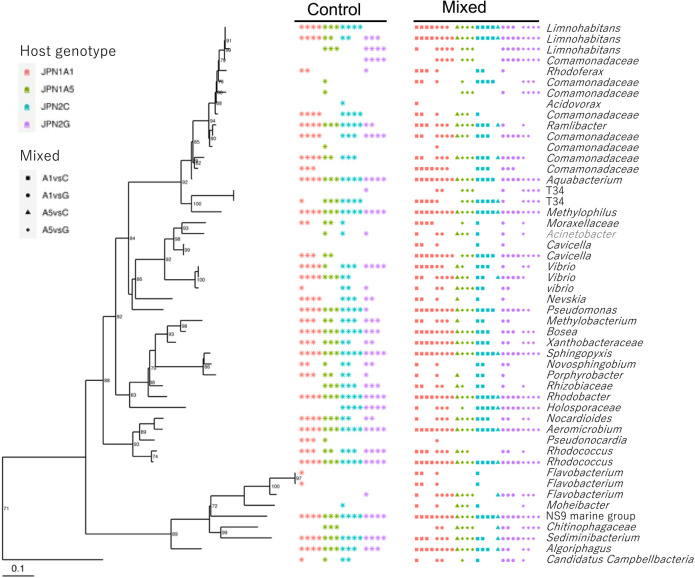
Phylogenetic tree of bacterial ASVs detected in individuals of four *Daphnia* cf. *pulex* genotypes grown under control and mixed treatments. Different genotype combinations in the mixed treatment are represented by distinct symbols, and ASVs from different genotypes are indicated by different colors. The number of symbols denotes the number of host individuals in which each ASV was detected. Numbers on nodes indicate bootstrap values (only values >70% are shown). The genus- or family-level taxonomic assignment of each ASV is also shown in the right column.

A total of 1,222 ASVs were obtained from host *Daphnia* individuals, with read counts ranging from 26,049 to 40,740 per host individual (mean: 31,450 reads). The phylogenetic relationships among abundant ASVs are shown in Fig. 1, alongside the host genotypes in which these ASVs were detected. ASVs belonging to genera such as *Limnohabitans*, *Aquabacterium*, *Methylophilus*, *Pseudomonas*, *Bosea*, *Sphingopyxis*, *Rhodobacter*, *Aeromicrobium*, *Rhodococcus*, NS9 marine group, *Sediminibacterium*, and *Algoriphagus* were consistently found across all host *Daphnia* genotypes, irrespective of treatment. In contrast, several undescribed ASVs exhibited treatment- and genotype-specific distributions. Undescribed ASVs of *Comamonadaceae*, *Flavobacterium* (ASV-1 and ASV-2), and an ASV affiliated with the order *Chitinophagaceae* were present exclusively in JPN1 genotypes under control conditions, but appeared in both the JPN1 and JPN2 genotypes in the mixed treatment. Conversely, ASVs belonging to *Horosporaceae*, *Flavobacterium* (ASV-3), and *Moheibacter* were specific to the JPN2 genotypes in control treatments, but occurred in both lineages under mixed conditions.

To compare within-host ASV diversity among *Daphnia* genotypes, we quantified α-diversity using the Shannon index for all ASVs. The JPN1 genotypes exhibited a Shannon index of approximately 2 in both the control and mixed treatments, with no significant difference between treatments (Fig. S1). JPN2 genotypes showed slightly lower α-diversity than JPN1, and this difference was not significant within either treatment group.

#### Relative abundance of host-associated bacteria

**Fig. 2.**
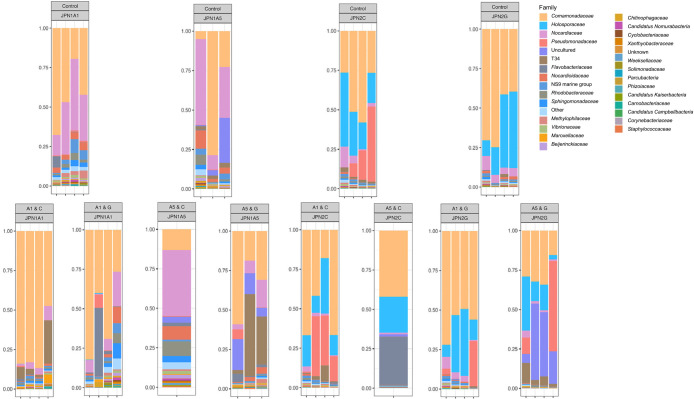
Relative abundance of bacterial taxa identified from individuals of four *Daphnia* cf. *pulex* genotypes (JPN1-A1, JPN1-A5, JPN2-C, and JPN2-G) grown with individuals of the same genotype (upper panels) or with different genotypes (lower panels). Bacterial taxa were grouped at the family level, except for those unclassified at this level, which were grouped at the order or class level. Only the top 30 taxonomic groups based on ASV read counts are shown.

We exami­ned differences in the relative abundance of the top 30 most abundant host-associated bacterial ASVs at the family level across all *Daphnia* host individuals (Fig. 2). Members of the family *Comamonadaceae* were the most dominant, followed by *Holosporaceae*. Although ASVs belonging to *Holosporaceae* were abundantly detected in both JPN2 genotypes, they were entirely absent in the control treatments of JPN1 genotypes and only rarely detected in mixed treatments of these genotypes. ASVs from the families *Nocardiaceae* and T34 were prevalent in JPN1 genotypes under both the control and mixed treatments; however, their relative abundance was lower in JPN2 genotypes. ASVs from *Pseudomonadaceae* were present in all host genotypes, while their relative abundance was consistently higher in JPN2 genotypes, regardless of treatment. In mixed treatments, *Flavobacteriaceae* ASVs were abundant in genotype A1 of the JPN1 lineage and genotype C of the JPN2 lineage. ASVs ranked below the top 30 in relative abundance did not exhibit clear differences across treatments or host genotypes.

We further categorized ASVs by their occurrence: 11 were unique to JPN1 genotypes, 2 to JPN2 genotypes, and 23 were shared between both lineages. Their relative abundances were compared between control and mixed treatments (Fig. S2). According to the Wilcoxon rank-sum test, no significant differences were observed between treatments for ASVs that were specific to either the JPN1 or JPN2 genotypes. However, for ASVs found in both lineages, significant differences in relative abundance between treatments were detected in JPN1 genotypes for *Rhodococcus*, *Flavobacterium*, *Aeromicrobium*, *Limnohabitans*, *Cavicella*, and *Ramlibacter*. In JPN2 genotypes, significant differences were observed for *Aeromicrobium*, *Chitinophagales*, *Limnohabitans*, *Sediminibacterium*, *Cavicella*, and *Nevskia*.

### β-Diversity

**Fig. 3.**
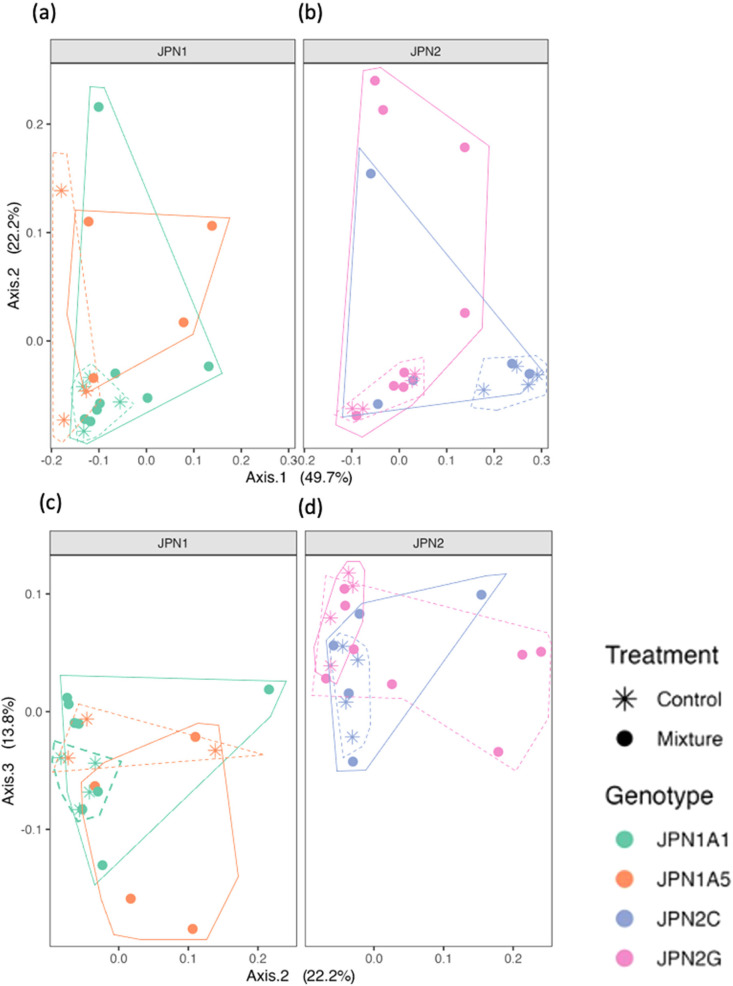
Biplots of first and second axes (a, b) and second and third axes (c, d) from PCoA anal­yses of β-diversity (weighted UniFrac distance, WUD) for host-associated microbiota of four *Daphnia* cf. *pulex* genotypes (JPN1-A1, JPN1-A5, JPN-2C, and JPN-2G) grown with individuals of the same genotype (control treatment) or different genotypes (mixed treatment). Different colors represent the microbiota of different host genotypes, while different symbols and line types indicate treatments.

**Fig. 4.**
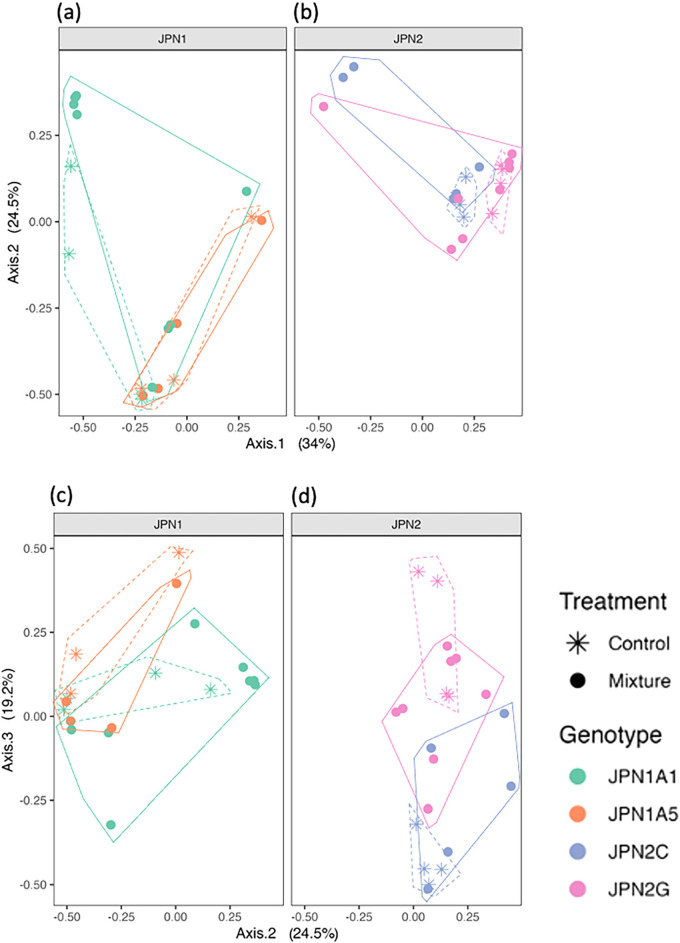
Biplots of first and second axes (a, b) and second and third axes (c, d) from PCoA anal­yses of β-diversity (Horn index) for host-associated microbiota of four *Daphnia* cf. *pulex* genotypes (JPN1A1, JPN1A5, JPN2C, and JPN2G) grown with individuals of the same genotype (control treatment) or different genotypes (mixed treatment). Different colors represent the microbiota of different host genotypes, and different symbols indicate treatments.

To assess compositional differences in microbiota among host genotypes under different treatments, PCoA was conducted using β-diversity metrics: WUD (Fig. 3) and the Horn index (Fig. 4). The first three PCoA axes accounted for 85.7 and 77.7% of the total variation in microbiota composition in WUD and Horn index anal­yses, respectively. In WUD-based PCoA, microbiota compositions in the control treatments showed low variations among individuals within the same host genotype and were partially separated between different host genotypes along the first axis (Fig. 3). Similarly, in the Horn index anal­ysis, the control treatment microbiota was clearly separated among host genotypes along the first and third axes (Fig. 4). In contrast, microbiota compositions in the mixed treatments displayed greater variability among individuals of the same genotype in both anal­yses, indicating a higher degree of individual-level divergence under mixed conditions.

**Fig. 5.**
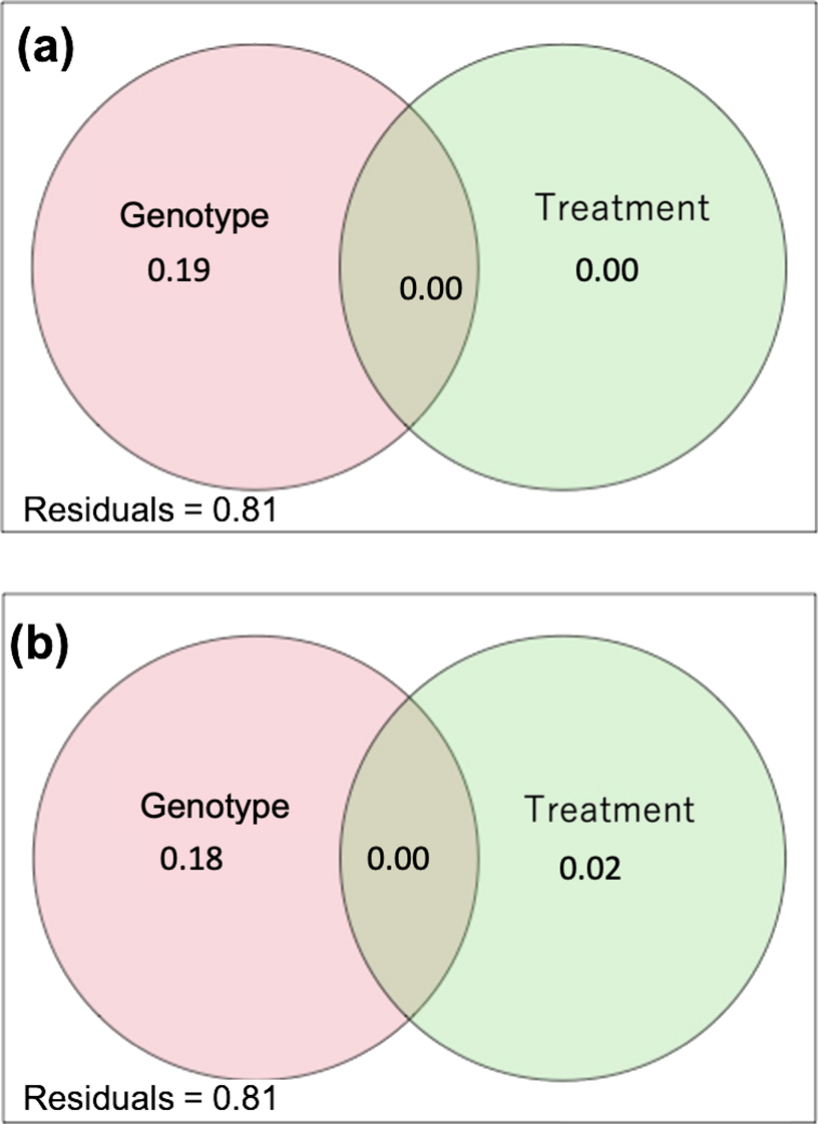
Results of variation partitioning showing the effects of genotype and experimental treatment on the ASV composition of host-associated microbiota, based on weighted UniFrac distance (WUD; a) and the Horn index (b). Contributions of genotype, treatment, their interaction, and residuals are shown in each panel.

A nested PERMANOVA revealed that β-diversity measured by WUD did not significantly differ between the control and mixed treatments. However, significant differences were observed between host lineages and between genotypes within lineages (Table 3). In contrast, β-diversity assessed using the Horn index showed significant differences between treatments, regardless of lineage or genotype (Table 3). As with the WUD anal­ysis, Horn index-based β-diversity also significantly differed between lineages and among genotypes within lineages, independent of treatment. The variation partitioning anal­ysis showed that host lineage uniquely explained 19 and 18% of variations in microbiota compositions in the WUD and Horn index anal­yses, respectively. Treatment (control vs. mixed) explained 0 and 2% of variations, respectively (Fig. 5).

## Discussion

In the present study, we found that the taxonomic composition of host-associated microbiota significantly differed between hosts grown with the same genotype and those co-cultured with a different genotype, as evaluated using the Horn index. Specifically, some ASVs belonging to the family *Comamonadaceae*, the genus *Flavobacterium*, and the order *Flavobacteriales* were absent from *Daphnia* cf. *pluex* JPN2 hosts when reared alone in the control treatment, but appeared when these hosts were co-cultured with JPN1 individuals in the mixed treatments. Conversely, ASVs of the family *Horosporaceae*, the genus *Flavobacterium*, and the genus *Moheibacter* were absent from JPN1 hosts in isolation, but were detected when co-cultured with JPN2 hosts. These results suggest that certain host-associated bacteria were horizontally transferred between genetically distinct hosts. In terrestrial animals, the horizontal transfer of gut microbes may occur through cannibalism, mating, sexual harassment, and other social interactions (*e.g.*, Tzuri *et al.*, 2021). The horizontal transmission of host-associated bacteria has also been documented in aquatic organisms, including fish and invertebrates (Sipkema *et al.*, 2015; Sylvain and Derome, 2017). In aquatic ecosystems, such bacteria may drift or disperse through the surrounding water after detachment from their hosts. Therefore, the horizontal transmission of host-associated bacteria is likely common in aquatic animals, including *Daphnia*, even in the absence of direct physical or social contact.

However, the effects of the horizontal transmission of bacteria from hosts of other lineages on host-associated microbiota did not appear to be deterministic. Although the taxonomic composition of host-associated microbiota was generally similar among hosts of the same genotype in the control treatment, it varied widely among these hosts when they were co-cultured with genotypes from different lineages. Unlike growing with the same genotype, cohabiting with genetically distinct hosts may have acted as a perturbation to host-associated microbiota. Recent studies demonstrated that even when microbiota were initially similar between hosts, stochastic bacterial interactions and subtle differences in host physiological state may lead to divergent microbiota communities (Jones *et al.*, 2022; Zaoli and Grilli, 2022). The horizontal transmission of host-associated bacteria likely accompanied these processes, resulting in greater variability in microbiota compositions among hosts of the same genotype in the mixed treatment.

Although it occurred, horizontal transmission was not sufficiently strong to affect the overall composition of host-associated microbiota. In the control treatments, where hosts were reared with the same genotype, taxonomic compositions significantly differed between genotypes within lineages and also between lineages. Moreover, the number of taxa did not change when *Daphnia* individuals were co-cultured with different lineage hosts in the mixed treatments. Although the Horn index detected some compositional differences between the control and mixed treatments, no significant differences were found when using WUD, which account for phylogenetic relatedness among bacterial taxa. These results indicate that although genetically distinct hosts sharing the same habitat may affect each other’s microbiota through horizontal transmission, this effect is insufficient to override the genotype-specific microbial community structure associated with each host.

The host-associated microbiota of *D.* cf. *pulex* may be primarily shaped by priority effects, as defined by De Meester *et al.* (2002) and Fukami (2015), rather than by the host’s genetic background. A recent study demonstrated that manipulating the immigration order of symbiotic bacteria in a *Daphnia* species often resulted in markedly different microbiota compositions, suggesting the impact of priority effects on the assembly of host-associated microbiota (Gurung *et al.*, 2024). In the present study, we used neonates less than 24 h old as hosts, meaning that the initial composition of their microbiota was likely affected for a short period by parental individuals. Once established, the resident microbiota likely inhibits colonization by most exogenous bacteria because symbionts that have adapted to the host environment efficiently occupy available niches. Consequently, the phylogenetic composition of host-associated microbiota within a *D.* cf. *pulex* genotype was not markedly affected, even when individuals were co-cultured with genetically distinct lineages. The *D.* cf. *pulex* genotypes used herein were collected from different lakes at different times and maintained for several years under constant laboratory conditions (Tian *et al.*, 2024). Nevertheless, host-associated microbiota significantly differed between the two lineages in all treatments, indicating that microbiota compositions in this species do not occur by chance. Although we cannot exclude a role for priority effects in shaping the microbiota observed in this study, differences in host genotypes appear to play an equally important, or more fundamental, role in establishing host-associated microbiota.

The taxonomic composition of host-associated microbiota, including the gut microbiota, is known to be shaped by both host genetic characteristics and environmental factors, such as water temperature and food quantity and quality (Dorosz *et al.*, 2016; Akbar *et al.*, 2022). However, the relative importance of these factors in *Daphnia* species remains somewhat controversial. Previous studies reported that environmental changes produce greater variations in host-associated microbiota compositions than differences among host genotypes (Hegg *et al.*, 2021; Eckert *et al.*, 2021). In contrast, other studies provided strong evidence to show that microbiota compositions are genetically constrained in *Daphnia* (Sullam *et al.*, 2018; Rajarajan *et al.*, 2023). Rajarajan *et al.* (2023) attributed the large variation observed in the *D. galeata* microbiota to the inclusion of a single unique genotype, suggesting that the effect size of genetic constraints depends on the degree of genetic and phenotypic divergence among the genotypes exami­ned. Supporting this inference, the present study demonstrated that genetic constraints played a large role when genetically distant genotypes were compared within the same species.

The genetic distance between the JPN1 and JPN2 lineages is markedly greater than that between genotypes within these lineages because they originated from independent introductions from North America into Japan at different times (So *et al.*, 2015; Ohtsuki *et al.*, 2022). These lineages also differ in several ecologically important traits (Tian *et al.*, 2024). Consequently, the host-associated microbiota may consistently differ between two lineages, even when they are reared together. While our results highlight the importance of genetic constraints on host-associated microbiota, they may underestimate the roles of horizontal transmission and environmental effects because this study was conducted under a single dietary and temperature regime. Certain environmental conditions may enhance the horizontal transmission of symbiotic bacteria even between genetically distant hosts, thereby reducing differences in the taxonomic composition of their microbiota.

Houwenhuyse *et al.* (2021) recently reported that some bacteria were consistently shared between two different *Daphnia* species, regardless of growth conditions or host genotype. Similarly, we identified several bacterial taxa that‍ ‍were present in all the host individuals across all experimental treatments, including *Limnohabitans*, *Aquabacterium*, *Methylophilus*, *Rhodobacter*, *Aeromicrobium*, and *Rhodococcus*. Although we used axenically cultured algae, the presence of most of these bacterial taxa appeared to be closely associated with the algal diet. However, previous studies showed that at least *Limnohabitans* is not‍ ‍dependent on algal diets (Ichige and Urabe, 2023) and‍ ‍may promote the growth of several *Daphnia* species (Peerakietkhajorn *et al.*, 2015; Zhang *et al.*, 2021). In contrast, Eckert *et al.* (2021) exami­ned multiple zooplankton species and found no evidence of core bacteria that characterize a particular species or taxon. Since the present study did not compare different *Daphnia* species, it remains unclear whether species-specific bacteria exist. However, we detected members of the family *Holosporaceae* only in JPN2 lineage genotypes, suggesting that they represent host lineage–specific bacteria. Given the abundance of environmentally or diet-associated bacteria, detecting genotype- or species-specific bacteria may be challenging.

There are several limitations that need to be addressed. *Daphnia* individuals were exami­ned within 8 days of growth, making it unclear whether the microbiota of hosts in the mixed treatment eventually revert to the composition observed in the control treatments or develop into a distinct, age-dependent state. It is also important to note that JPN1-A1 and JPN2-G were collected from the same lake (Lake Hataya Ohnuma) and maintained under identical laboratory conditions. Nevertheless, their host-associated microbiota differed to some extent, suggesting that the microbiota composition was at least partially affected by host genetic characteristics; however, it was not possible to assess the magnitude of this effect in the present study. In addition, because only two genetically distant lineages were exami­ned, it remains unclear whether the rate of horizontal transmission between hosts is affected by their genetic relatedness. This transmission may occur less frequently between more distantly related genotypes or lineages. Addressing these uncertainties in future studies will be essential for disentangling the relative contributions of genetic and environmental factors to the assembly of host-associated microbiota in aquatic animals.

## Citation

Ichige, R., and Urabe, J. (2026) Host Genetic Constraints on the Horizontal Transmission of *Daphnia*-associated Microbiota. *Microbes Environ ***41**: ME26003.

https://doi.org/10.1264/jsme2.ME26003

## Supplementary Material

Supplementary Material

## Figures and Tables

**Table 1.** Number of replications and number of host individuals inoculated per bottle in each treatment.

Treatment	No. of replications	Genotype	No. of individuals	Genotype	No. of individuals
Control	2	JPN1-A1	4		
Control	2	JPN1-A5	4		
Control	2	JPN2-C	4		
Control	2	JPN2-G	4		
Mixed	3	JPN1-A1	2	JPN2-C	2
Mixed	3	JPN1-A1	2	JPN2-G	2
Mixed	3	JPN1-A5	2	JPN2-C	2
Mixed	3	JPN1-A5	2	JPN2-G	2

**Table 2.** Number of individuals used in genetic and microbiota anal­yses. Control treatments are denoted by bold letters and mixed treatments by italic letters.

Genotype		Genotype of co-individuals				Total
		JPN1-A1	JPN1-A5	JPN2-C	JPN2-G	
JPN1 lineage						
	JPN1-A1	**4**		*4*	*4*	12
	JPN1-A5		3	1	3	7
JPN2 lineage						
	JPN2-C	*4*	*1*	**4**		9
	JPN2-G	*4*	*1*		**4**	12

**Table 3.** Results of PERMANOVA on effects of host lineage, host genotype within the lineage, and the experimental treatment on the β-diversity of host-associated microbiota, measured using weighted UniFrac distances.

Factors	df	Weighted UniFrac distance			Horn Index	
		Sum of square	F		Sum of square	F
Host lineage	1	0.306	12.31 ***		2.594	11.68 ***
Treatment	1	0.031	1.23		0.523	2.36 *
Host lineage : Genotype	2	0.176	3.54 **		2.059	4.64 ***
Host lineage : Treatment	1	0.059	2.37		0.345	1.55
Host Lineage : Genotype : Treatment	2	0.089	1.79		0.367	0.83
Residual	32	0.796			7.109	
Total	39	1.457			12.998	

*, *P*<0.05; **, *P*<0.01; and ***, *P*<0.001
