# Supplementary material for: Host Genetic Constraints on the Horizontal Transmission of Daphnia-associated Microbiota
Source: Microbes Environ. 2026 May 27;41(2):ME26003. doi: 10.1264/jsme2.ME26003 (PMC13293707; doi:10.1264/jsme2.ME26003)
Supplement: Supplementary file 1 — Supplementary Material [file 41_26003_s1.pdf]

Supplementary Material

Host Genetic Constraints on the Horizontal Transmission of Daphnia-associated Microbiota

Ryotaro Ichige, Jotaro Urabe

Figure S1.
Figure S2.

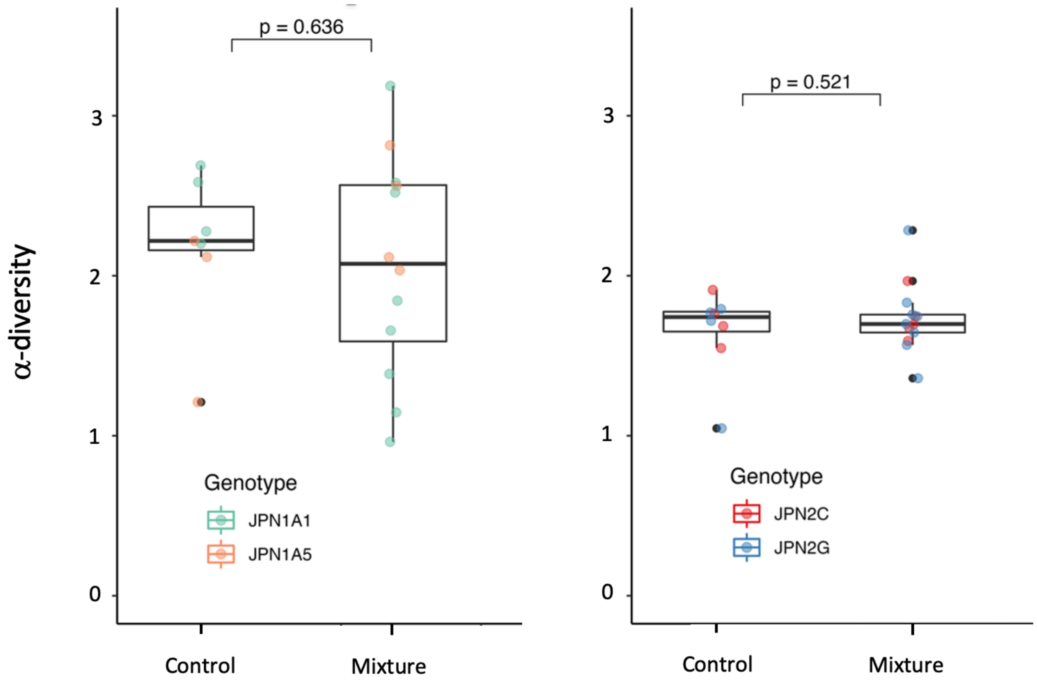

Figure S1. Box plots showing the 25th, 50th, and 75th percentiles, and the 95% confidence limits of the mean α-diversity (Shannon index) of host-associated microbiota in the JPN1 lineage (a) and JPN2 lineage (b). Hosts were grown with individuals of the same lineage (control treatment) or a different lineage (mixed treatment). The α-diversity of each individual is indicated by symbols in different colors for different genotypes. *p*-values from *t*-tests comparing control and mixed treatments are shown in each panel.

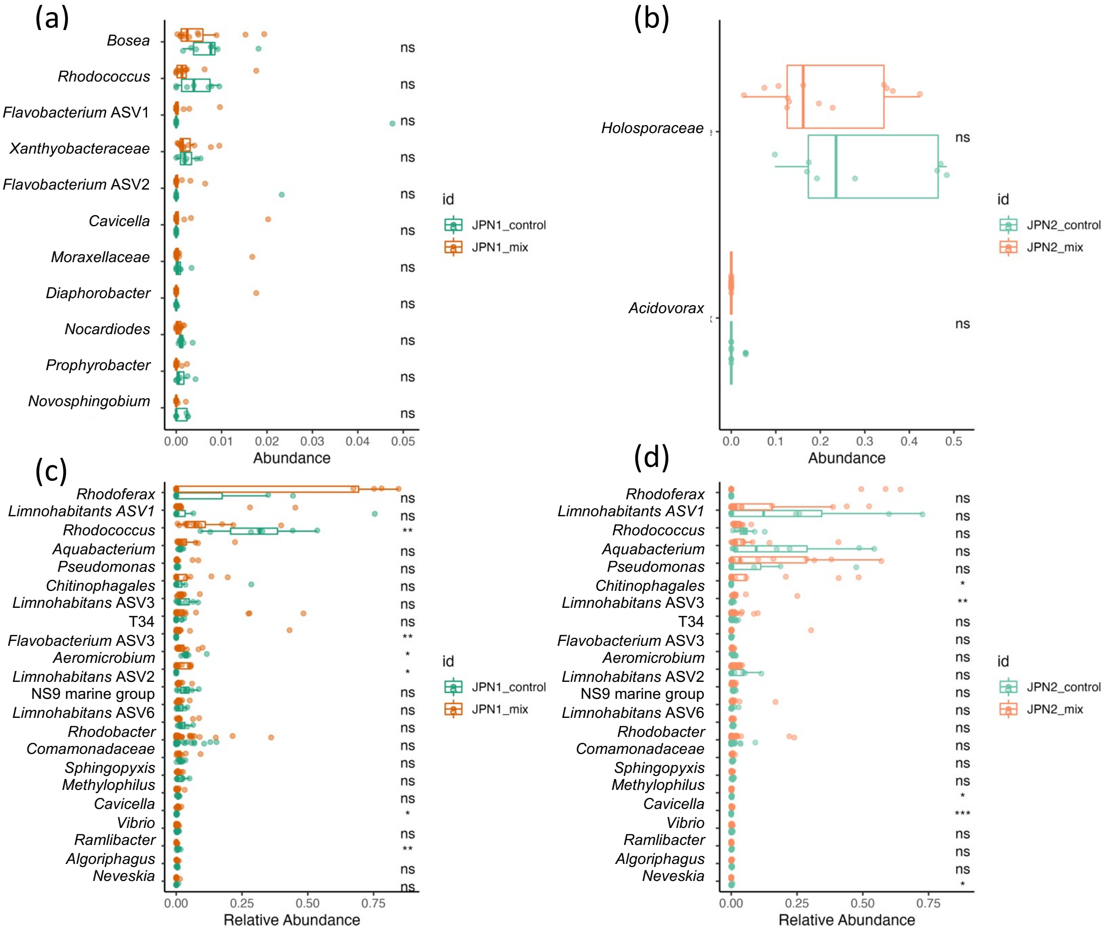

Figure S2. Relative abundance of ASV specific to JPN1 lineage (a) or JPN2 lineage (b) but commonly found in individuals grown in the control and mixed treatments, and that of ASV commonly found in JPN1 lineage (c) and JPN2 lineage (d). Significant difference in the relative abundance between individuals grown in the control and mixed treatments examined by Wilcoxon rank sum test is indicated in each panel (*: $p<0.05$, **: $p<0.01$; and ***: $p<0.001$).
